# Performance Enhancement of Ultra-Thin Nanowire Array Solar Cells by Bottom Reflectivity Engineering

**DOI:** 10.3390/nano10020184

**Published:** 2020-01-21

**Authors:** Xin Yan, Haoran Liu, Nickolay Sibirev, Xia Zhang, Xiaomin Ren

**Affiliations:** 1 State Key Laboratory of Information Photonics and Optical Communications, Beijing University of Posts and Telecommunications, Beijing 100876, China; xyan@bupt.edu.cn (X.Y.); lhrbyr@bupt.edu.cn (H.L.); xmren@bupt.edu.cn (X.R.); 2 ITMO University, St. Petersburg 197101, Russia; nicksibirev@yandex.ru

**Keywords:** nanowire, solar cell, bottom reflectivity, conversion efficiency

## Abstract

A bottom-reflectivity-enhanced ultra-thin nanowire array solar cell is proposed and studied by 3D optoelectronic simulations. By inserting a small-index MgF_2_ layer between the polymer and substrate, the absorption is significantly improved over a broad wavelength range due to the strong reabsorption of light reflected at the polymer/MgF_2_ interface. With a 5 nm-thick MgF_2_ layer, the GaAs nanowire array solar cell with a height of 0.4–1 μm yields a remarkable conversion efficiency ranging from 14% to 15.6%, significantly higher than conventional structures with a much larger height. Moreover, by inserting the MgF_2_ layer between the substrate and a part of the nanowire, in addition to between the substrate and polymer, the absorption of substrate right below the nanowire is further suppressed, leading to an optimal efficiency of 15.9%, 18%, and 5.4% for 1 μm-high GaAs, InP, and Si nanowire solar cells, respectively. This work provides a simple and universal way to achieve low-cost high-performance nanoscale solar cells.

## 1. Introduction

In recent years, new styles of solar cells, such as organics, perovskites, dye sensitization, and nanowires (NWs), have made great progress [1,2,3,4]. For example, over 15% efficiency was obtained for a single-junction organic solar cell, and even higher efficiency was expected for a tandem architecture [1,5]. Among those structures, semiconductor NWs have unique advantages in stability and lifetime. In comparison with its planar counterpart, standing NWs are superior in theory due to the very low filling ratio, strong light trapping, and light-concentrating ability, as well as the weak emission of photons [6,7,8,9]. Moreover, the ultra-small footprint area of NWs significantly increases the tolerance of lattice mismatch, enabling the realization of low-cost solar cells on cheap substrates and high-performance tandem solar cells [10,11,12,13]. To date, vertical NW array (NWA) solar cells, based on different materials, have been demonstrated [6,14,15,16,17,18]. For example, the vertical InP and GaAs NWA solar cells with axial p-i-n junctions yielded an efficiency of 13.8% and 15.3%, with a low filling ratio of 12% and 13%, respectively [6,14]. However, the efficiency of NWA solar cells is the lack of competitiveness in comparison to their planar counterparts and because they still require further optimizations. In addition, the total height of the NW array also needs to be further compressed to reduce the material cost.

Thus far, many efforts have been made to improve the optical absorption of the NWA, which plays a key role in the photovoltaic performance of NW solar cells. One way to improve the optical absorption is to tailor the structural parameters, including the diameter, diameter/period (D/P) ratio, geometry, and orientation of the NWA [19,20,21,22,23]. Another way is to introduce other nanostructures, such as semiconductor quantum dots (QDs) or metal nanoparticles, to extend the absorption spectrum or to enhance the absorption efficiency [24,25]. These methods typically lead to an absorption enhancement at certain wavelengths, or in a narrow wavelength range. In addition to the absorption enhancement of the NWA itself, the reduction of the incident light waste, such as the absorption by substrate, is also a promising way to increase the absorption efficiency. Polymers have been widely used in optoelectronic devices, and in a practical NWA solar cell, polymers are typically filled among NWs for electrical insulation and are supporters of the top electrode [26,27,28]. As the polymer typically has a much smaller refractive index in comparison to that of the III-V or Si substrate [29,30], a certain amount of light would be absorbed by the substrate, leading to a substantial waste of incident light. If the light incident upon the substrate is reflected and reabsorbed by the NWs, the total absorption of the NWs would be substantially enhanced and the corresponding photovoltaic performance would be significantly improved.

In this paper, we propose a p-i-n NWA solar cell with bottom reflectivity enhancement (BRE-NWA for short). The reflectivity enhancement is achieved by inserting a thin MgF_2_ layer between the polymer and substrate. As MgF_2_ has a lower refractive index compared to the polymer, the light incident upon the substrate is strongly reflected and reabsorbed by the NWs, leading to an absorption enhancement over the whole absorption spectra. The photovoltaic properties of practical devices are studied by a coupled three-dimensional (3D) opto-electronic simulation. The doping-dependent mobility, bandgap narrowing, and radiative, Auger, and SRH recombinations are all taken into consideration in the electrical simulations. The properties of the device without the MgF_2_ layer are also investigated for comparison. The results show that the MgF_2_ layer significantly improves the whole absorption of the NWs, leading to a remarkable enhancement in efficiency. Moreover, by inserting the MgF_2_ layer between the substrate and a part of NW, in addition to between the substrate and polymer, the absorption of substrate right below the NW is further suppressed, leading to an optimal efficiency of 15.9%, 18%, and 5.4% for GaAs, InP, and Si NWAs, respectively. The designs are particularly promising for self-powered integrated microsystems and space applications.

## 2. Methods

**Figure 1:**
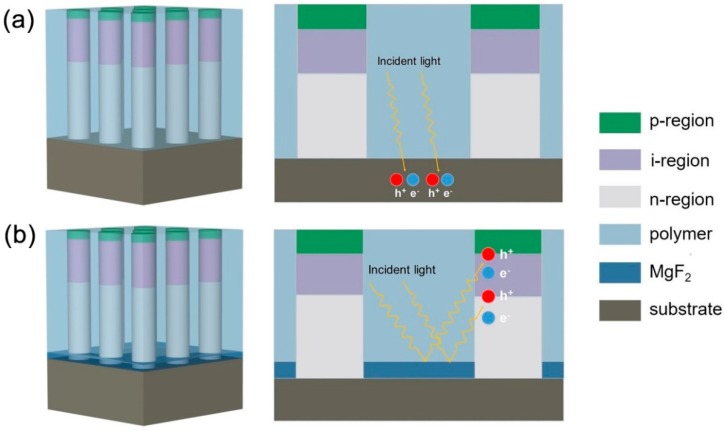
Schematic diagrams of the (**a**) nanowire arrays (NWA) and (**b**) bottom reflectivity enhancement (BRE-NWA) solar cell structures.

Figure 1 shows the schematic structures of devices studied in this work. A classical NWA solar cell is presented in Figure 1a, which consists of 1 μm high NWs capsulated in polymers (polymethyl methacrylate (PMMA) is adopted in this work) on a substrate with the same material as the NWs. Each NW contains an axial p-i-n junction, in which the length ratios of p, i, and n-regions are 1:5:9 [14]. The p and n-regions are uniformly doped to 1 × 10^18^ cm^−3^ and 3 × 10^18^ cm^−3^, respectively, and the GaAs substrate was n-doped with a carrier concentration of 3 × 10^18^ cm^−3^. In the NWA structure, a considerable amount of light striking the substrate will be absorbed by the substrate, leading to a substantial loss of incident light. Figure 1b shows the proposed BRE-NWA solar cell, in which a thin dielectric MgF_2_ was inserted between the polymer and substrate. As MgF_2_ has a lower refractive index compared to the polymer (the refractive index for MgF_2_ and polymer is ~1.38 and ~1.5, respectively, in the wavelength range from 290 to 900 nm), the light striking the substrate was strongly reflected and reabsorbed by the NWs, leading to an absorption enhancement. The fabrication process of the proposed BRE-NWA solar cell could be summarized as follows. After the MgF_2_ layer was deposited on an n-type GaAs substrate by the electron beam evaporation, periodic opening patterns were formed by electron-beam lithography, and by wet-chemical etching techniques. The patterned substrate was then loaded into a metalorganic vapor phase epitaxy system, and GaAs NWs were grown from the pitches via selective-area epitaxy [31,32,33]. The axial p-i-n structure was formed by growing the n, i, and p sections in turn. The n and p doping was realized by introducing Si and Zn dopants, respectively. After growth, the surface passivation was achieved by immersing the as-grown GaAs NWs in the nitride or sulfide solutions, reducing the surface recombination velocity (SRV) from 10^6^–10^7^ cm/s to ~10^3^ cm/s [34,35,36]. Then, the NWA was filled with polymer by spin casting, followed by ion etching to remove the polymer from the tip of the NWs. Transparent electrodes, such as indium tin oxides (ITOs), were deposited on the NW tips to form the top contacts. Depositing metals onto the substrate forms back contacts.

Optical properties of the structure were investigated through the Sentaurus Electromagnetic Wave (EMW) Solver module package. The minimum cell size of the finite-difference-time-domain (FDTD) mesh was set to 0.5 nm, and the number of nodes per wavelength in the x and y directions was 10, and in the z direction, it was 15. By placing periodic boundary conditions, the simulations could be carried out on a single unit cell to model the periodic array structure. The substrate thickness was set to 0.4 μm to save the resources and time required for the simulation. The wavelength-dependent complex refractive index used in the simulations was obtained from References [37,38,39]. We used a plane wave defined with power intensity and wavelength values from a discretized AM 1.5G solar spectrum to model the sunlight. The transverse electric (TE) and transverse magnetic (TM) mode contributions were superimposed to model the sunlight. The total optical generation under AM 1.5G illumination could be modeled by superimposing the power-weighted single-wavelength optical generation rates. The optical generation rate $G_{ph}$ was obtained from the Poynting vector S:

(1) $$G_{ph}=\frac{\left|\vec{\nabla }\cdot \vec{S}\right|}{2\hbar \omega }=\frac{\varepsilon^{\prime \prime }{\left|\vec{E}\right|}^{2}}{2\hbar }$$

where ℏ is the reduced Planck’s constant, ω is the angular frequency of the incident light, E is the electric field intensity at each grid point, and $\varepsilon^{\prime \prime }$ is the imaginary part of the permittivity. The reflection monitor was located above the top surface of the NWA, and the transmission monitor was located at the bottom surface of NWs to calculate the light absorbed by the NWs. The amount of power transmitted through the power monitors was normalized to the source power at each wavelength. The reflectance R(λ) and transmission T(λ) were calculated by the equation:

(2) $$R(\lambda ),T(\lambda )=0.5\int real\{p{\left(\lambda \right)}_{monitor}\}dS/P_{in}(\lambda )$$

where P(λ) is the Poynting vector, dS is the surface normal, and $P_{in}(\lambda )$ is the incident source power at each wavelength. The absorption spectrum A(λ) of the NWs was given by the following equation

(3) A(λ)=1−R(λ)−T(λ)

For the electrical modeling, the 3-D optical generation profiles were incorporated into the finite-element mesh of the NWs in the electrical tool, which solved the carrier continuity equations coupled with Poisson’s equation self-consistently in 3D. The doping-dependent mobility, bandgap narrowing, and radiative, Auger, and SRH recombination were taken into consideration in the device electrical simulations. In addition, due to the high surface/volume ratio, surface recombination played an important role in the photoelectric conversion, especially for GaAs, which typically had a high surface recombination velocity (SRV) of 10^6^–10^7^ cm/s. Fortunately, the SRV of GaAs NWs could be reduced to ~10^3^ cm/s by surface passivation [34,35,36]. In our structures, surface passivation could be realized by simply immersing the as-grown GaAs NW array in the nitride or sulfide solutions before spinning the polymer. Hence, in the studies, the NWs were assumed to be passivated with a SRV of 10^3^ cm/s.

## 3. Results and Discussion

**Figure 2:**
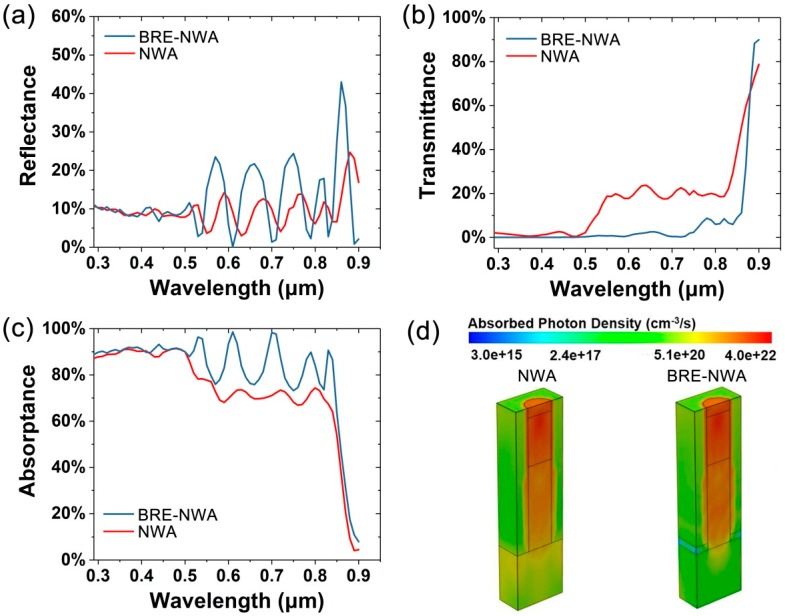
(**a**) Reflectance, (**b**) transmittance, and (**c**) absorptance of the NWA and BRE-NWA structures. (**d**) 3-D total optical generation profiles in the NWA and BRE-NWA structures.

Figure 2a–c present the wavelength-dependent reflectance, transmittance, and absorptance of the GaAs NWs in two structures shown in Figure 1. The diameter, length, and D/P ratio of NWs are set to 180 nm, 1 μm, and 0.5, respectively. The thickness of MgF_2_ layer is 50 nm. From the reflectance spectra, oscillations are clearly observed in the wavelength range from 500 to 900 nm in both structures. Those oscillations are attributed to the longitudinal modes along the length of the NW. Due to the large refractive index difference between the NW and its surroundings, a waveguide cavity is formed along the NW length. The reflectance peak and valley are described by the interference condition for longitudinal modes, $Ln_{eff}=m\lambda /4$, where L is the NW length, $n_{eff}$ is the effective refractive index of the NW array, m is an integer, and λ is the wavelength of incident light [40]. The spacing between resonances is approximated as $\Delta \lambda =\lambda^{2}/2Ln_{eff}$. As the NWs in both structures have the same length, the peak and valley wavelengths in the reflectance spectra are determined by the effective refractive index. As MgF_2_ has a smaller refractive index compared with the polymer, the effective refractive index of the BRE-NWA structure is smaller than the conventional NWAs, resulting in a blueshift of the reflectance peak and valley wavelengths, as shown in Figure 2a. For example, the two resonance peaks observed for NWA in Figure 2a (λ = 680 nm, 770 nm) correspond to $n_{eff}$ ~2.92, while the two resonance peaks (λ = 660 nm, 750 nm) for BRE-NWA correspond to $n_{eff}$ ~2.76. Moreover, the amplitude of the oscillations significantly increases after introducing the MgF_2_ layer, which is attributed to the reflectivity enhancement at the polymer/MgF_2_ interface. Figure 2b shows the transmittance spectra of the two NW array structures. The transmittance is reduced by about 20% after introducing an MgF_2_ layer in the wavelength range from 500 to 900 nm. For the conventional NWA structure, as the refractive index of GaAs substrate is much higher than the polymer, a portion of light is easy to pass through the polymer to the substrate and be absorbed. After introducing an MgF_2_ layer, most of the light striking the polymer/MgF_2_ interface will be reflected back, resulting in a much lower transmittance. Thanks to the strong light-trapping effect of vertical NWs, most of the reflected light will be reabsorbed by the NWs. Hence, after introducing an MgF_2_ layer, the absorptance of NWs is significantly enhanced, as shown in Figure 2c. A small portion of the reflected light, which is not absorbed by the NWs, will return to the air, contributing to the enhancement of reflectance at some wavelengths shown in Figure 2b. However, the light reabsorbed by the NWs is much more than that reflected to the air, resulting in a much higher absorption in spite of a slightly higher reflectance at partial wavelengths.

In order to analyze the absorption properties more intuitively, 3-D total optical generation profiles under AM 1.5G illumination in half of the structures are shown in Figure 2d. Clearly, for the conventional NWA structure, a considerable amount of light is absorbed by the substrate. After introducing the MgF_2_ layer, the absorption in the substrate is significantly suppressed. Only a little bit of light is absorbed by the substrate right below the NW due to the similar refractive index between the NW and substrate, which will be further suppressed in the following optimizations.

**Figure 3:**
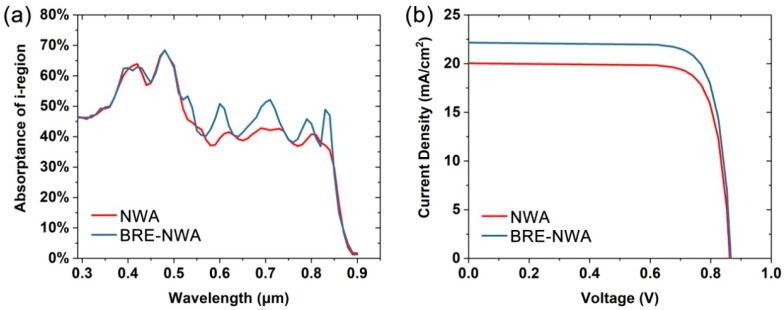
(**a**) Absorption spectra of the i-region and (**b**) current-voltage curves of the NWA and BRE-NWA structures.

For a practical p-i-n NWA solar cell, the absorption enhancement of the whole NW array does not directly lead to an increase in the ultimate conversion efficiency, as the photocarriers generated in the p (or n) region quickly recombine due to the lack of a built-in electric field. Instead, the absorption in the depletion region directly determines the ultimate efficiency to some degree. Figure 3a shows the integral of the absorption spectra in the i-region. The absorptance of each wavelength is weighted by the AM 1.5G spectrum. Evidently, the absorption in i-region is enhanced in the wavelength range from 500 to 900 nm after introducing the MgF_2_ layer, which will finally lead to an increase in conversion efficiency. The photogeneration profiles are then incorporated into the electrical tool to study the photovoltaic performance of the structures. Figure 3b shows the current-voltage characteristics of the two structures. It can be seen that the BRE-NWA structure yields a short-circuit current density ($J_{sc}$) of 22.2 mA/cm^2^ (2.2 mA/cm^2^ higher than the NWA structure) and open-circuit voltage ($V_{oc}$) of 0.805 V (similar to the NWA structure), resulting in a higher conversion efficiency (η) of 15.6% compared to 13.9% of the NWA structure. The results demonstrate that the introduction of a thin MgF_2_ layer is a simple and effective way to improve the conversion efficiency without changing the parameters of the NWA.

**Figure 4:**
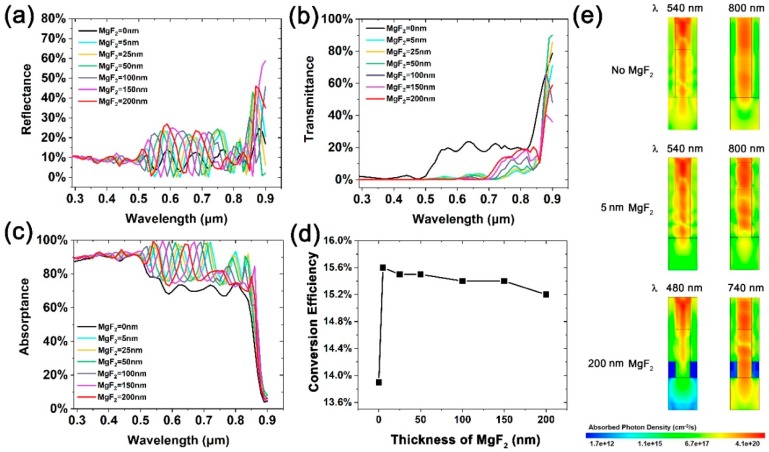
(**a**) Reflectance, (**b**) transmittance, (**c**) absorptance, and (**d**) conversion efficiency of BRE-NWA structures with different MgF_2_ thicknesses. (**e**) Vertical cross sections of optical generation profiles at several absorption peaks for 0, 5, and 200 nm thick MgF_2_ layers.

The thickness of the MgF_2_ layer has a significant influence on the light absorption of the NWA. On the one hand, the proportion between polymer and MgF_2_ dominates the effective refractive index of the NWA, which further influences the light field. On the other hand, the thickness of MgF_2_ also determines the reabsorption amount of the reflected light by the NWs. The MgF_2_–thickness-dependent reflectance, transmittance, and absorptance of the NWs are shown in Figure 4a–c. From the reflectance spectra, we can clearly see a blueshift of reflectance peak and valley wavelengths as the thickness of MgF_2_ increases, which is attributed to a decreasing effective refractive index of the NWA. In addition, the amplitude of the reflectance oscillations increases at shorter wavelengths and decreases at longer wavelengths when increasing the MgF_2_ thickness, in accordance with the transmittance that decreases shorter wavelengths and increases at longer wavelengths, as shown in Figure 4b. From the absorptance spectra shown in Figure 4c, we can see that all the BRE-NWA structures exhibit significantly higher absorption, regardless of the MgF_2_ thickness. The absorptance is similar for different MgF_2_ thicknesses in the wavelength range from 300 to 750 nm, except for a blueshift of absorption peak wavelength by increasing the MgF_2_ thickness. At longer wavelengths beyond 750 nm, the absorption obviously decreases as the MgF_2_ thickness increases, leading to a slight degradation in the photovoltaic performance, as shown in Figure 4d. As the MgF_2_ thickness increases from 5 to 200 nm, the conversion efficiency gradually decreases from 15.6% to 15.2%. One reason for the efficiency degradation is the decrease in the effective refractive index of the whole NWA, which leads to a weaker confinement of light in the NW waveguides. For example, the two resonance peaks for 50 nm-thick MgF_2_ layers observed in Figure 4a (λ = 660 nm, 750 nm) correspond to $n_{eff}$ ~2.76, while the two resonance peaks for 200 nm-thick MgF_2_ layers (λ = 590 nm, 680 nm) correspond to $n_{eff}$ ~2.24. Figure 4e shows the vertical cross sections of optical generation profiles at the absorption peak around 500 and 800 nm, with an MgF_2_ thickness of 0, 5, and 200 nm, respectively. It can be seen that the light intensity inside the NW decreases as the MgF_2_ thickness increases. In addition, for short wavelength light, the absorption maximum occurs at the upper part of NW. As the wavelength increases, the absorption extends downward to the lower part. As the MgF_2_ thickness has little effect on the reflected light striking the upper part of NW, the absorption at short wavelengths changes a little. However, the increased MgF_2_ thickness causes more long-wavelength light confined in the NW bottom and transmitted into the substrate due to a larger refractive index difference between the NW and MgF_2_, resulting in increased transmittance at long wavelengths. Meanwhile, the light reflected by the MgF_2_ layer is reduced, leading to an absorption drop at long wavelengths, particularly the absorption in the i-region. The results demonstrate that an ultra-thin 5 nm MgF_2_ layer is enough to promote the conversion efficiency, while an MgF_2_ layer that is too thick leads to a degradation of the performance, in addition to an increase in cost.

**Figure 5:**
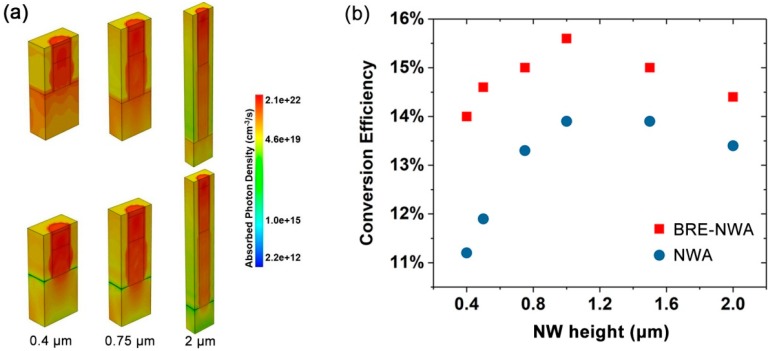
(**a**) 3-D total optical generation profiles in the NWA and BRE-NWA structures with a NW height of 0.4, 0.75, and 2 μm, respectively. (**b**) Conversion efficiency comparison of NWA and BRE-NWA structures with different NW heights.

As the high-reflectivity-layer significantly enhances the absorption of the NW array, it is possible to achieve high-performance solar cells with ultra-short NWs to further reduce the material cost. Figure 5a shows the 3-D total optical generation profiles under AM 1.5G illumination in half of the structures with different NW lengths of 0.4, 0.75, and 2 μm, respectively. For the 0.4 μm-high NWA, a great deal of light is absorbed by the substrate due to the insufficient absorption of the short NWs. As the NW height increases, the light transmitted into the substrate decreases, and the absorption in the NW increases. However, even at a relatively large height of 2 μm, the absorption by the substrate is still relatively strong. After introducing the MgF_2_ layer, the absorption by the substrate is significantly suppressed for all NW heights. The reduction of absorption in the substrate and enhancement of absorption in the NW are particularly apparent for the shortest NWA, which are attributed to the insufficient absorption of the incident light, and strong reabsorption of the reflected light by the short NWs. The NW length-dependent conversion efficiency of NWA and BRE-NWA structures is shown in Figure 5b. The efficiency of both structures first increases and then decreases as the NW height increases, which is attributed to the insufficient absorption for short NWs and enhanced recombination of photo-generated electron-hole pairs for long NWs. Nevertheless, all the BRE-NWA structures exhibit much higher efficiency than the conventional NWAs. Even at an ultra-small height of 0.4 μm, the BRE-NWA structure yields a remarkable efficiency of 14%, which is higher than the conventional NWAs with a much larger height, enabling the realization of ultra-thin, low-cost solar cells. It should be mentioned that other nanostructures, such as QDs grown by the Stranski–Krastanov method, have even smaller heights in comparison to NWs. While QDs have shown advantages in having low-thresholds and high-temperature-stability light emission devices, strain-induced dislocations in QDs lead to considerable non-radiative recombinations, limiting the short-circuit current and conversion efficiency [41,42]. By contrast, NWs typically possess high crystal quality, which are more suited for light conversion applications.

**Figure 6:**
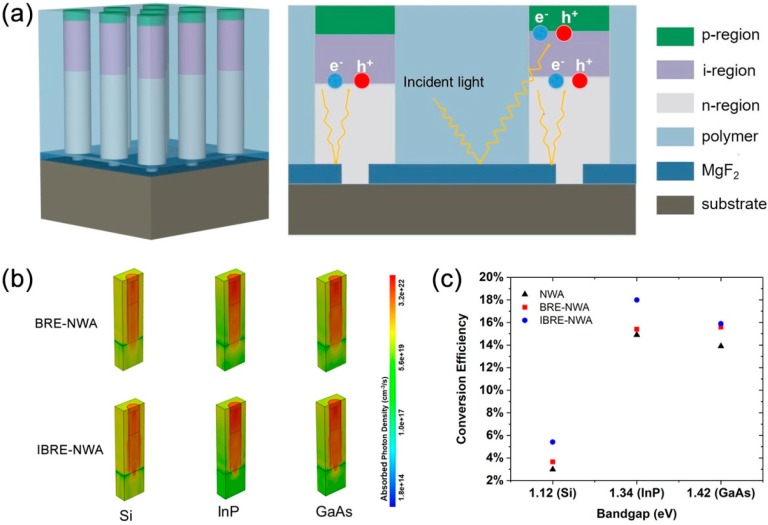
(**a**) The schematic diagram of the IBRE-NWA structure. (**b**) 3-D total optical generation profiles in the BRE-NWA and IBRE-NWA structures. (**c**) Conversion efficiency comparison of NWA, BRE-NWA, and IBRE-NWA structures.

From Figure 2d and Figure 5a, we can clearly see the phenomenon that strong absorption still occurs in the substrate right below the NW, which is attributed to the similar refractive index between the NW and substrate. To further suppress this part of absorption, the structure is further optimized by inserting the MgF_2_ layer between the substrate and a part of the NW, in addition to between the substrate and polymer, as shown in Figure 6a. In the improved structure (IBRE-NWA for short), a considerable amount of light striking the NW/substrate interface will be strongly reflected by the MgF_2_ layer and be reabsorbed by the NW. The fabrication process of the IBRE-NWA is similar to the BRE-NWA, except for the NW growth. For the IBRE-NWA structure, the size of the mask opening is smaller than that of the BRE-NWA structure, resulting in thinner NWs in the initial growth stage. After growing a short, thin NW higher than the MgF_2_ layer, the growth is switched from axial to radial mode, resulting in a mushroom-like morphology. Then, the growth is switched back from radial to axial mode to complete the subsequent growth. To demonstrate the universality of the photovoltaic enhancement of the IBRE-NWA structures, other semiconductor materials are also investigated. We chose InP, which is suitable for the solar spectrum with a direct band gap of 1.34 eV, and Si, the most commonly used material in solar cells in present studies. Figure 6b presents the 3-D total optical generation profiles in half of the structures for GaAs, InP, and Si BRE-NWA and IBRE-NWA structures. It can be seen that apparently the absorption right below the NW is significantly suppressed by the MgF_2_ layer for all the three materials. Figure 6c shows the conversion efficiency of NWA, BRE-NWA, and IBRE-NWA structures in GaAs, InP, and Si materials, respectively. With a bottom NW (the part inserted in the MgF_2_) diameter of 70 nm, the conversion efficiency of the GaAs, InP, and Si IBRE-NWA structures reaches 15.9%, 18%, and 5.4%, respectively, which is higher than the reported results with the same material, as shown in Table 1. The results demonstrate the universal applicability of the bottom-reflectivity layer in the performance enhancement of the NWA solar cells.

## 4. Conclusions

In conclusion, we propose and study a bottom-reflectivity-enhanced vertical NWA solar cell. By inserting a small-index MgF_2_ layer between the substrate and polymer among NWs, the absorption of NW array is enhanced over a broad wavelength range, which is due to the strong reabsorption of light reflected at the polymer/MgF_2_ interface. Moreover, by inserting the MgF_2_ layer between the substrate and a part of the NW, in addition to between the substrate and polymer, the absorption of the substrate right below the NW is further suppressed, leading to an optimal efficiency of 15.9%, 18%, and 5.4% for GaAs, InP, and Si NWA solar cells, respectively, significantly higher than conventional NWA structures. The low-cost high-performance devices are particularly promising for self-powered microsystems and space applications.

## Figures and Tables

**Table 1:** Photovoltaic performance of reported p-i-n junction NWA solar cells.

Material	$V_{oc}\;(V)$	$J_{sc}\;(\mathrm{mA}/{\mathrm{cm}}^{2})$	Fill Factor (%)	Efficiency (%)	Reference
GaAs	0.868	22.7	80.5	15.9	This work
GaAs	0.906	21.3	79.2	15.3	[14]
GaAs	0.565	21.08	63.65	7.58	[28]
InP	0.835	24.9	86.3	18	This work
InP	0.779	24.6	72.4	13.8	[6]
InP	0.73	21	73	11.1	[43]
Si	0.559	11.8	82.3	5.4	This work
Si	0.45	6.34	53	1.47	[44]
Si	0.29	4.28	33	0.46	[15]
